# Low FODMAP Diet Relieves Visceral Hypersensitivity and Is Associated with Changes in Colonic Microcirculation in Water Avoidance Mice Model

**DOI:** 10.3390/nu15051155

**Published:** 2023-02-24

**Authors:** Chenmin Hu, Chenxi Yan, Yuhao Wu, Enfu Tao, Rui Guo, Zhenya Zhu, Xiaolong Chen, Marong Fang, Mizu Jiang

**Affiliations:** 1 Endoscopy Center and Gastrointestinal Laboratory, Children’s Hospital, Zhejiang University School of Medicine, National Clinical Research Center for Child Health, National Children’s Regional Medical Center, Hangzhou 310052, China; 2 Department of Pediatrics, Affiliated Hangzhou First People’s Hospital, Zhejiang University School of Medicine, Hangzhou 310006, China; 3 Children’s Hospital, Zhejiang University School of Medicine, National Clinical Research Center for Child Health, Hangzhou 310052, China; 4 Institute of System Medicine, Zhejiang University School of Medicine, Hangzhou 310058, China; 5 Department of Gastroenterology, Children’s Hospital, Zhejiang University School of Medicine, National Clinical Research Center for Child Health, National Children’s Regional Medical Center, Hangzhou 310052, China

**Keywords:** colonic microcirculation, irritable bowel syndrome, FODMAP, diet, visceral hypersensitivity

## Abstract

(1) Background: Irritable bowel syndrome (IBS) is a global public health problem, the pathogenesis of which has not been fully explored. Limiting fermentable oligosaccharides, disaccharides, monosaccharides, and polyols (FODMAP) can relieve symptoms in some patients with IBS. Studies have shown that normal microcirculation perfusion is necessary to maintain the primary function of the gastrointestinal system. Here, we hypothesized that IBS pathogenesis might be related to abnormalities in colonic microcirculation. A low-FODMAP diet could alleviate visceral hypersensitivity (VH) by improving colonic microcirculation; (2) Methods: C57BL/6 mice were raised to establish an IBS-like rodent model using water avoidance (WA) stress or SHAM-WA as a control, one hour per day for ten days. The mice in the WA group were administered different levels of the FODMAP diet: 2.1% regular FODMAP (WA-RF), 10% high FODMAP diet (WA-HF), 5% medium FODMAP diet (WA-MF), and 0% low FODMAP diet (WA-LF) for the following 14 days. The body weight and food consumption of the mice were recorded. Visceral sensitivity was measured as colorectal distention (CRD) using the abdominal withdrawal reflex (AWR) score. Colonic microcirculation was assessed using laser speckle contrast imaging (LCSI). Vascular endothelial-derived growth factor (VEGF) was detected using immunofluorescence staining; (3) Results: The threshold values of CRD pressure in the WA-RF, WA-HF, and WA-MF groups were significantly lower than those in the SHAM-WA group. Moreover, we observed that colonic microcirculation perfusion decreased, and the expression of VEGF protein increased in these three groups of mice. Interestingly, a low-FODMAP dietary intervention could reverse this situation. Specifically, a low-FODMAP diet increased colonic microcirculation perfusion, reduced VEGF protein expression in mice, and increased the threshold of VH. There was a significant positive correlation between colonic microcirculation and threshold for VH; (4) Conclusions: These results demonstrate that a low-FODMAP diet can alter VH by affecting colonic microcirculation. Changes in intestinal microcirculation may be related to VEGF expression.

## 1. Introduction

Irritable bowel syndrome (IBS) is a functional gastrointestinal disease with a global impact [1,2], affecting 3.8–4.8% of the general population [3,4]. Chronic, recurrent abdominal pain associated with an altered stool form or frequency is the clinical characteristic of IBS [5]. Currently, the understanding of the complex pathogenesis of IBS is limited. It is believed to be related to microbiota-brain-gut axis communication disorder [6,7], visceral hypersensitivity (VH) [8], gastrointestinal infection [9], digestive tract inflammation [10], and brain function changes [11].

Research has shown that dietary manipulation is critical in managing IBS, especially fermentable oligosaccharides, disaccharides, monosaccharides, and polyols (FODMAP) [12]. FODMAP is an acronym for “Fermentable Oligosaccharides, Disaccharides, Monosaccharides, and Polyols”. It exists in a variety of different and common foods. Different foods contain different kinds of FODMAP, for instance, fruits, vegetables, milk, legumes, honey, and sweeteners. Some foods contain only one kind, while others contain several kinds.

FODMAP is poorly absorbed or nonabsorbed in healthy individuals [13], so excessive small molecular substances increase the osmotic activity in the intestinal cavity, forcing water to enter the gastrointestinal tract and expand the intestinal cavity. When the unabsorbed FODMAP enters the colon, it becomes the substrate for the fermentation of gas-producing bacteria, producing many gases, such as hydrogen, methane, and short-chain fatty acids [13,14]. Excessive gas further worsens the expansion of the intestinal cavity and triggers IBS clinical symptoms in a dose-dependent manner [15]. Recently, a study showed that lactose and fructo-oligosaccharides could raise colonic glycosylation end-product production and increase visceral sensitivity in mice [16]. In addition, it is also believed that the fermentation metabolites of anaerobic bacteria, especially alcohol, ketone, and aldehyde, can affect the calcium signal and lead to the imbalance of intestinal flora, which may also be the cause of IBS [17]. In clinical practice, it is common for IBS to combine with small intestinal bacterial overgrowth (SIBO). Several symptoms are shared by both SIBO and IBS, including abdominal pain, distention, diarrhea, and bloating.

A high FODMAP diet can aggravate intestinal inflammation, damage the intestinal epithelial barrier, induce mast cell activation, and finally trigger characteristic IBS clinical symptoms, such as abdominal pain, bloating, and fecal urgency [18,19,20]. Randomized controlled clinical studies and preclinical studies have revealed that a low FODMAP diet can alleviate symptoms in some cases but not in all patients with IBS, which is superior to other interventions [21,22], although there are still many controversies [23]. The FODMAP can activate mast cells and release more inflammatory mediators, for example, histamine, which can affect the immune system [16,24]. In addition, the FODMAP diet can be sensitive to nociceptive neurons and the intestinal nervous system [25]. Furthermore, it was proved that dysfunction of the gut-brain axis is responsible for the generation ofsymptoms caused by FODMAP [26].

Microcirculation refers to blood circulation through microvessels that are less than 100 µm in diameter, including arterioles, capillaries, and venules. It plays a critical role in the human body by delivering oxygen to adjacent tissues, providing energy, and maintaining organ homeostasis. Additionally, microcirculation mediates immune system activity and hemostasis [27]. The primary function of the gastrointestinal system is to digest and absorb nutrients and prevent the invasion of bacteria and microorganisms, which intrinsically requires normal microcirculation perfusion level [28,29]. Numerous mechanisms can regulate vascular tension in gastrointestinal circulation, primarily by the nervous system, hormones, and metabolic mechanisms [29].

Over the last two decades, intestinal microcirculation has attracted increasing attention. Advanced techniques for evaluating microcirculation are developing rapidly, including intravital microscopy, laser speckle contrast imaging (LSCI), laser doppler flowmetry, and optical coherence tomography. Alexander et al. found that the incidence of ischemic colitis in patients is 3.4 times higher than that in those without IBS [30]. However, the relationship between IBS and colon ischemia remains to be established. In addition, changes in colonic microcirculation after FODMAP diet intervention still need to be investigated.

In the present study, we investigated the changes in colonic microcirculation in an animal model of IBS and after the intervention with different FODMAP diets using a direct and intuitive technique. Moreover, we analyzed critical protein signaling pathways in intestinal vasculogenesis and angiogenesis to understand the changes in potential colonic microcirculation mechanisms.

## 2. Materials and Methods

### 2.1. Animals and Water-Avoidance Procedures

#### 2.1.1. Animals

Adult male C57/BL6 mice (20–22 g) were purchased from SLAC Laboratory Animal Co., Ltd. (Shanghai, China). The animals were raised in polypropylene cages with a light-dark cycle (light was turned off at 9:00 am, turned on at 9:00 pm, and then turned off automatically). Each cage housed 4–6 animals at a humidity of 50 ± 5% and a room temperature of 25–26 °C. Food and water were provided ad libitum. The animal research ethics committee approved the animal research protocol of Zhejiang University.

#### 2.1.2. Dietary Formula

The mice were fed a custom-made diet (Wuxi Fanbo Biotechnology Co., Ltd., Wuxi, China) of regular FODMAP (RF) during the period of chronic water avoidance (WA) stress (Table 1). They were then randomly divided into four groups and continued to receive RF, high FODMAP diet (HF), medium FODMAP diet (MF), and low FODMAP diet (LF) (Table 1) [18]. The diet intervention lasted for 14 days [31]. Mice in the sham water-avoidance group received an RF diet.

The diets were designed to mimic human dietary consumption of FODMAP (Table 1). Comparing the composition of the raw materials between the groups, the quality differences of fructose, fructo-oligosaccharide, and galacto-oligosaccharide are the most critical. The difference was designed to be regulated by the quality of corn starch, another type of carbohydrate, such that the final weight ratio and energy ratio of protein, fat, and carbohydrate among the groups remained the same.

#### 2.1.3. WA Stress Protocol and Grouping

**Figure 1:**
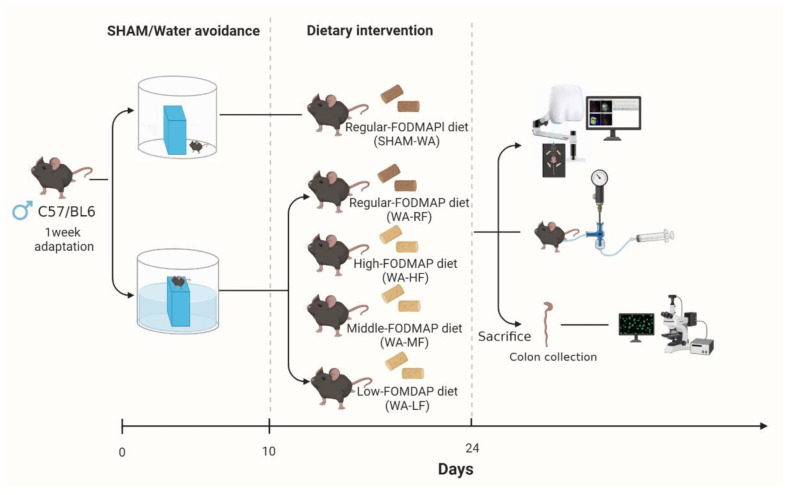
Schematic diagram of the experimental protocol.

Similar to the previous experiment [32,33], the customized test device included a transparent plastic tank (45 cm length × 32 cm width × 26 cm height) and a cuboid acrylic (3 cm length × 3 cm width × 9 cm height) fixed at the bottom center. The tank was filled with fresh room temperature water (25 °C) up to 1 cm below the acrylic bar. This was done according to the chronic WA stress protocol for one hour per day for ten consecutive days. The experimental animals were divided into SHAM-WA, WA-RF, WA-HF, WA-MF, and WA-LF groups, with 8–9 animals in each group (Figure 1).

### 2.2. Visceral Hypersensitivity Measurement

In mice, the abdominal withdrawal reflex (AWR) score was assessed using colorectal distension (CRD). We invented a novel and exquisite distension balloon (length 1 cm, diameter 0.5 cm), which was published previously [34]. After 30 min, the mice adapted to the environment. The balloon was lubricated with Vaseline, inserted into the rectum for 1 cm, and appropriately fixed under 2% isoflurane (RWD Life Science, Shenzhen, China) anesthesia. In the first stage, gas was continuously injected into the balloon slowly, the reaction of the mice was observed, and the corresponding pressure value was recorded. In the second stage, the balloon was promptly inflated to constant pressure (on the order of 10, 20, 30, 40, 50, 60, 70, 80, and 90 mmHg pressure), and the AWR value of the mouse colorectum under different pressures was measured. AWR scoring standard was set as 0: no response; 1: slight head movement and no body movement; 2: abdominal muscle contraction; 3: abdominal uplift; 4: body arch-back elevation. The AWR score was measured by two independent observers using a double-blind method. Each experiment was repeated thrice to obtain an average value, and the mice were allowed to rest for 15–20 min between the 2 stages.

### 2.3. Immunofluorescence Staining

Frozen sections of 8 μm thickness were dried at 37 °C in an oven, then washed twice with phosphate-buffered saline (PBS) for 10 min each time, and twice with 0.3%Triton (Triton X-100, FuDebio Science, Hangzhou, China) for 10 min each time. After blocking with 2% bovine serum albumin (A1933, Sigma Aldrich, Saint Louis, MO, USA) for 1 h at room temperature, the sections were incubated with primary antibodies (anti-VEGF CAT#A0280, ABclonal, Wuhan, China) at 4 °C overnight. Next, the sections were washed with PBST 4 times for 10 min each and incubated with secondary antibodies (1:1000, Dylight594, Goat anti-rabbit, FuDebio Science) for 1.5 h at room temperature. After that, the sections were washed with PBST 4 times, 10 min each time, and PBS 2 times, 5 min each time in sequence. Antifade mounting medium with 4′,6-diamidino-2-phenylindole (DAPI, Coolaber, Beijing, China) was added to the sections, and the slides were covered. Fluorescence signals were observed using a fluorescence microscope (Olympus VS200, Tokyo, Japan) under a 20× objective. The results were quantified using ImageJ software.

### 2.4. Laser Speckle Contrast Imaging

Colonic microcirculation blood flow was assessed directly using LSCI [35,36,37] (Laser Speckle Imaging system; RFLSI III; RWD Life Science, Shenzhen, China). Before initiating the study, parameters were corrected according to the manufacturer’s recommendations. An approximate infrared laser source with a wavelength of 785 nm was used to irradiate the surface of the tissue of interest, with a depth of 1 mm. The movement of particles in tissues (such as red blood cells) leads to speckle patterns randomly [38]. The sliding spatial mode with a high signal-to-noise ratio was adopted, with a 5 ms exposure time. The average microcirculatory blood flow (mFlux) results of the region of interest (ROI) were recorded using a CMO camera (image resolution 2048 × 2048-pixel, 10 frames/s) for 10 s. Finally, the mean mFlux within the ROI was calculated using the laser speckle blood flow imaging system v5.0.

The specific procedures were as follows: after isoflurane gas anesthesia was administered, the mice were placed in a supine position on a black background, the limbs were fixed with double-sided adhesive tape, the abdominal hair was removed with an electric razor, and a longitudinal incision was cut in the midline. All the intestinal segments were completely exposed. On the premise of not changing the original position of the mouse intestinal tube, each intestinal segment of the mouse was skillfully identified. To maintain moisture, exposed intestinal segments must be coated with normal saline. The laser probe was fixed 15 cm above the area to be measured. mFlux of the proximal colon was measured, and then the intestine was unfolded into a fan shape, and the mFlux of the proximal colon and the mesentery of the colon was investigated again.

### 2.5. Statistic Analyses

Statistical analyses were performed using SPSS23.0 (IBM, Armonk, NY, USA) and GraphPad Prism9.0 (GraphPad Software Inc., San Diego, CA, USA). All data are expressed as the mean ± SEM. Verifying that the original data had a normal distribution, body weight, food intake, and CRD measurements were analyzed using two-way ANOVA followed by Sidak’s multiple comparison tests. The remaining results were analyzed using a one-way ANOVA followed by Bonferroni’s post-hoc test. Correlation coefficients (r) were calculated using Pearson’s correlation coefficient. *p* < 0.05 was considered statistically significance.

## 3. Results

### 3.1. Body Weight Change and Dietary Consumption

**Figure 2:**
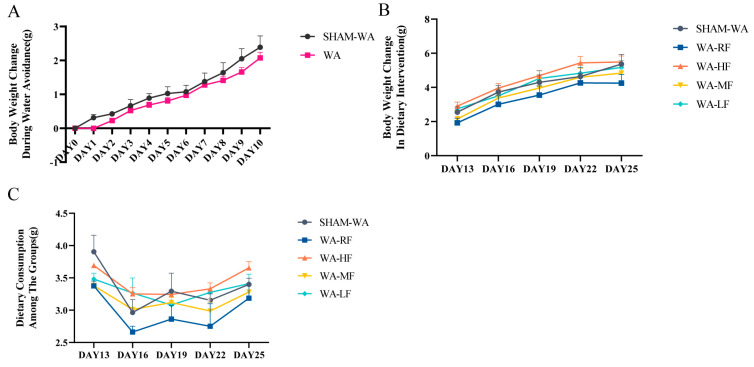
Body weight change and dietary consumption in mice. (**A**) Comparison of body weight change between SHAM−WA and WA groups during the WA period. There was no significant difference in body weight between the SHAM−WA and WA groups. (**B**,**C**) The weight changes of body weight and dietary consumption among groups every three days after different levels of FODMAP diet intervention. There was no significant difference in body weight changes and dietary consumption among the five groups. WA, Water avoidance; RF, regular FODMAP; HF, high FODMAP; MF, medium FODMAP; LF, low FODMAP. *n* = 8~9. Data were expressed as mean ± SEM by two-way ANOVA followed by Sidak’s multiple comparisons tests.

We observed fluctuations in the weight of the mice during WA. The weight of DAY0 is the baseline, and DAYn-DAY0 expresses the weight change. The results showed no statistical difference between the control and model groups during the WA stress (Figure 2A). During the diet intervention, body weight and feed consumption were measured every three days. Average food consumption = (original amount − residual amount)/number of days/number of mice in each cage. The results showed no significant differences in body weight and average feed consumption among the groups (Figure 2B,C).

### 3.2. Visceral Hypersensitivity

**Figure 3:**
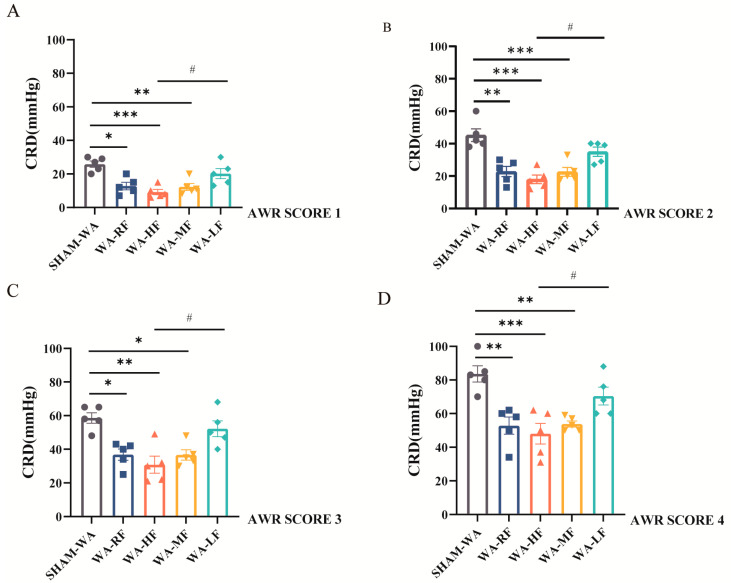
CRD threshold for AWR of different scores level. (**A**) The CRD threshold of each groupat AWR SCORE 1. (**B**) The CRD threshold of each group at AWR SCORE 2. (**C**) The CRD threshold of each group at AWR SCORE 3. (**D**) The CRD threshold of each group at AWR SCORE 4. AWR, abdominal withdrawal reflex; CRD, colorectal distension; WA, Water avoidance; RF, regular FODMAP; HF, high FODMAP; MF, medium FODMAP; LF, low FODMAP. *n* = 5. Data were expressed as mean ± SEM by two-way ANOVA followed by Sidak’s multiple comparisons tests. * *p* < 0.05, ** *p* < 0.005, *** *p* < 0.0005 versus SHAM-WA group, # *p* < 0.05 versus WA-LF group.

At an abdominal withdrawal test score of 1 point, the threshold values of colorectal distension pressure in the SHAM-WA, WA-RF, WA-HF, WA-MF, and WA-LF groups were 25.80 ± 1.88, 12.80 ± 2.22, 9.20 ± 1.59, 12.26 ± 2.03 and 20.20 ± 3.02 mmHg, respectively (Figure 3A); at a score of 2 points, the threshold values in each group were 45.20 ± 3.93, 22.80 ± 3.18, 18.00 ± 2.63, 22.60 ± 2.73 and 35.00 ± 2.88 mmHg, respectively (Figure 3B); at a score of 3 points, the threshold values in each group were 58.60 ± 3.14, 36.80 ± 3.34, 30.80 ± 5.03, 36.60 ± 3.08 and 52.20 ± 4.72, respectively (Figure 3C); at a score of 4 points, the thresholds of each group were 83.60 ± 4.84, 52.80 ± 5.12, 48.00 ± 6.14, 53.80 ± 1.83 and 70.40 ± 5.31 mmHg, respectively (Figure 3D). At the AWR1-4 score level, the thresholds of the WA-RF, WA-HF, and WA-MF groups were significantly lower than those of the SHAM-WA group, whereas the WA-LF group had a statistically significant difference.

**Figure 4:**
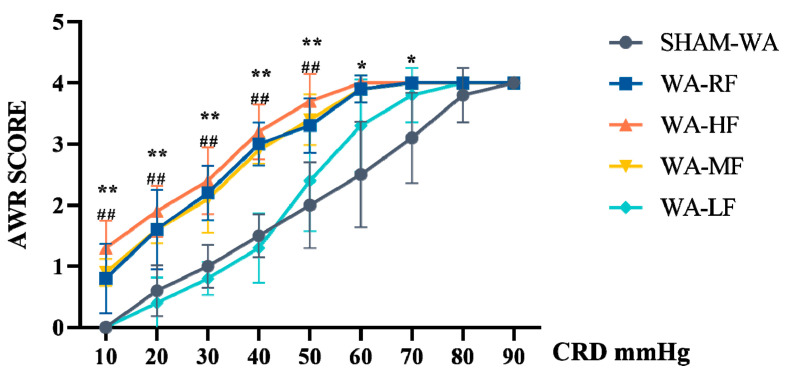
The AWR scores were evaluated among SHAM-WA, WA-RF, WA-HF, WA-MF, and WA-LF groups, under the constant pressure of 10–90 mmHg. There was a statistically significant difference at 10, 20, 30, 40, 50, 60, and 70 mmHg between SHAM-WA and WA-HF, and at 10, 20, 30, 40, and 50 mmHg between WA-HF and WA-LF. AWR, abdominal withdrawal reflex; CRD, colorectal distension; WA, Water avoidance; RF, regular FODMAP; HF, high FODMAP; MF, medium FODMAP; LF, low FODMAP. *n* = 5. Data was expressed as mean ± SEM by one-way ANOVA by Bonferroni’s Post-test. * *p* < 0.05, ** *p* < 0.0001, versus SHAM-WA group, ## *p*< 0.0001 versus WA-LF group.

Under the pressure of 10–90 mmHg order, the AWR scores in the SHAM-WA, WA-RF, WA-HF, WA-MF, and WA-LF groups were evaluated. The data were analyzed using two-way ANOVA (diet × pressure) followed by Sidak’s multiple comparisons test, interaction: F(32, 180) = 240.8, *p* < 0.0001. It had a significant main effect on dietary intervention: F(8, 180) = 240.8, *p* < 0.0001; moreover, it also had a significant main effect on pressure: F(4, 180) = 68.20, *p* < 0.0001. There was no significant difference at 80 and 90 mmHg between SHAM-WA and WA-HF and at 60, 70, 80, and 90 mmHg between WA-HF and WA-LF (Figure 4).

### 3.3. Colonic Microcirculation Blood Flow Decreased in the WA-RF, WA-HF, and WA-MF Groups, and Was Reversible in the WA-LF Group

Due to physiological hypoxia in healthy individuals, intestinal blood flow is vulnerable to microcirculation disorders and intestinal ischemia [39]. Laser speckle blood flow is a powerful noninvasive tool [40], which can be used as a visual method to dynamically measure changes in intestinal and mesenteric microcirculation blood flow in vivo [41]. This study presents the first data on the use of live real-time imaging tools in mice to focus on the changes in colon blood flow in IBS model mice and FODMAP diet intervention.

**Figure 5:**
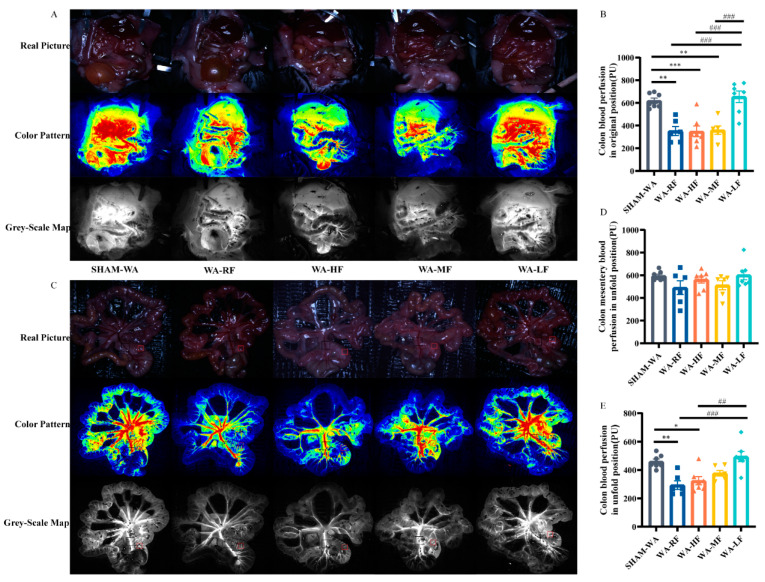
The representative pictures of colonic microcirculation perfusion in real-time and the statistical results. (**A**) The representative pictures of colonic microcirculation perfusion in the original position and (**B**) Thestatistical histogram of colonic blood perfusion in original position. (**C**) The representative pictures of colonic and mesenteric microcirculation perfusion in a fan-shaped position and (**D**,**E**) The statistical histogram of colonicmesentery perfusion and colonic blood perfusion in afan-shaped position. RF, regular FODMAP; HF, high FODMAP; MF, medium FODMAP; LF, low FODMAP; *n* = 6–7. Data was expressed as mean ± SEM by one-way ANOVA by Bonferroni’s Post-test. * *p* < 0.05, ** *p* < 0.005, *** *p* < 0.0005 versus SHAM-WA group, ## *p* < 0.005, ### *p* < 0.0005 versus WA-LF group.

In the original position, the results of the colonic mFlux in SHAM-WA, WA-RF, WA-HF, WA-MF, and WA-LF groups were 618.97 ± 24.03, 352.04 ± 40.07, 348.32 ± 46.73, 359.80 ± 36.69, and 654.66 ± 50.70 Perfusion Unit (PU) (Figure 5A,B). In the fan-shaped position, the results of mesenteric vessels mFlux were 594.74 ± 15.14, 575.23 ± 81.09, 561.79 ± 30.39, 512.20 ± 39.95, and 602.25 ± 42.25; the results of the colonic mFlux in SHAM-WA, WA-RF, WA-HF, WA-MF, and WA-LF groups were 460.22 ± 17.87, 293.84 ± 29.88, 324.47 ± 29.06, 377.45 ± 19.87, and 495.01 ± 35.94 (Figure 5C–E). The results showed that the colonic mFlux in the WA-RF, WA-HF, and WA-MF groups was lower than that in the SHAM-WA group in the original and fan-shaped positions. The low-FODMAP diet intervention could reverse this situation. There was no significant difference in the mFlux of the mesenteric vessels supplying the colon among the groups. Therefore, the decrease in colonic microcirculation blood flow perfusion after WA stress and FODMAP diet intervention may have been caused by local colon lesions.

### 3.4. VEGF Fluorescence Intensity Increased in the WA-HF Group and Decreased in the WA-LF Group

**Figure 6:**
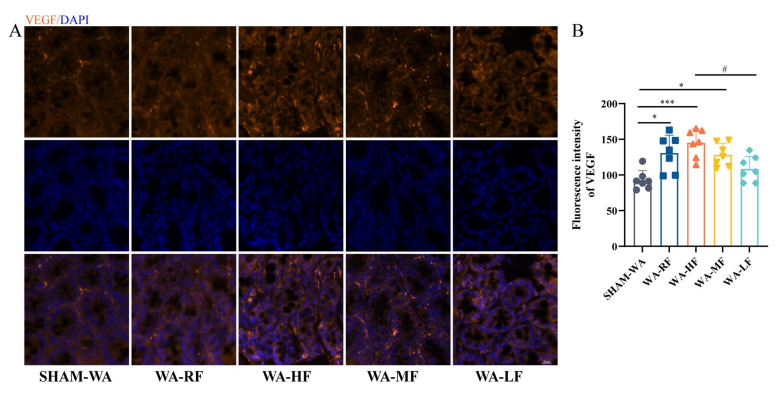
The immunofluorescence intensity of VEGF protein between groups. It was higher in the WA-RF, WA-HF, and WA-MF groups, compared to the SHAM-WA group and WA-HF group. (**A**) The representative pictures of VEGF protein expression in the colonic submucosa layer, and (**B**) The statistical results. VEGF, vascular endothelial-derived growth factor; DAPI, 2-(4-Amidinophenyl)-6-indolecarbamidine dihydrochloride; RF, regular FODMAP; HF, high FODMAP; MF, medium FODMAP; LF, low FODMAP; *n* = 7. Data was expressed as mean ± SEM by one-way ANOVA by Bonferroni’s Post-test. * *p* < 0.05, *** *p* < 0.0005 versus SHAM-WA group, # *p*< 0.05 versus WA-LF group.

We observed the colonic submucosa layer, which accounts for 70% of the intestinal blood flow, and the immunofluorescence intensity of VEGF protein in the SHAM-WA, WA-RF, WA-HF, WA-MF, and WA-LF groups were 92.75 ± 5.03, 130.83 ± 9.39, 145.01 ± 7.37, 128.06 ± 6.11, 108,48 ± 6.65. The result showed that the expression of VEGF in the WA-RF, WA-HF, and WA-MF groups was significantly higher than that in the SHAM-WA group. However, the level in the WA-LF group was significantly lower than that in the WA-HF group (Figure 6A,B).

### 3.5. Colonic Microcirculation Blood Flow Was Positively Correlated with the Threshold of VH

**Figure 7:**
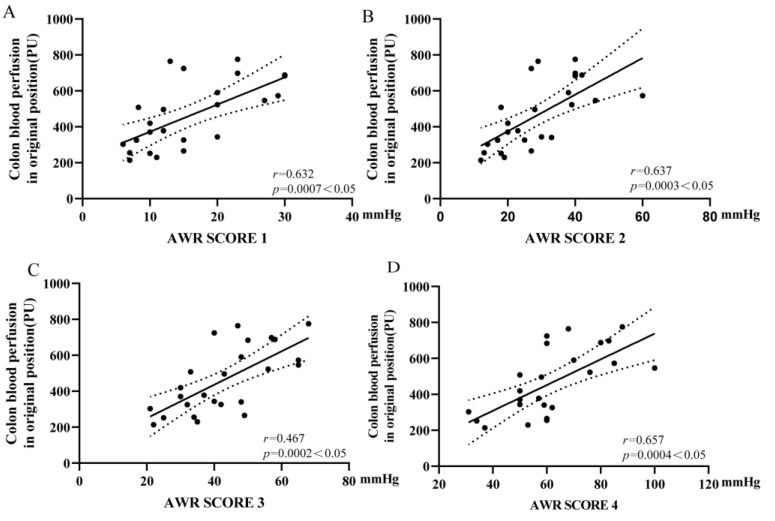
Scatter plot analysis of the correlation between visceral hypersensitivity and colonic microcirculation blood flow. (**A**–**D**) AWR score was 1–4, colonic microcirculation blood correlated significantly with the threshold of CRD (r = 0.632, 0.637, 0.467 and 0.657, *p* = 0.0007, 0.0003, 0.0002 and 0.0004 respectively).

When the AWR scores were 1, 2, 3, or 4, there was a positive correlation between colonic microcirculation blood flow and the threshold of VH, with 95% confidence bands displayed by dashed lines (r = 0.632, 0.637, 0.467, and 0.657; *p*= 0.0007, 0.0003, 0.0002, and 0.0004, respectively) (Figure 7A–D).

## 4. Discussion

In this study, we provided evidence that regular FODMAP, high FODMAP, and middle FODMAP diet interventions can aggravate VH, while low FODMAP can alleviate WA stress in mice. In addition, colonic microcirculation blood flow decreased in the WA-RF, WA-HF, and WA-MF groups, leading to colonic microcirculation dysfunction, while the expression of VEGF protein increased. Moreover, we found that a low-FODMAP diet can increase colonic blood perfusion and reduce VEGF protein expression.

There is evidence that diet is closely related to the balance of intestinal microecology. Dietary restriction may be essential in altering microbiota composition in people with IBS. However, the results are inconsistent. Some studies demonstrated that compared with low FODMAP and high FODMAP intervention, there was no difference in *α*-diversity and *β*-diversity [42]. In contrast, other studies showed that the total abundance of low FODMAP decreased [43]. Short-term restriction of the FODMAP diet can increase the abundance of *Bacteroides*, *Firmicutes*, *Clostridium*, and *Actinobacteria* while the abundance of *Bifidobacteria* decreases [44,45]. Among them, Bacteroides is a kind of bacteria related to the degradation of sugars [46].

VH is one of the most critical characteristics of IBS pathogenesis [47]. VH for mechanical stimulation of the colon is common among patients with IBS. Utilizing the rectal balloon distention method, evidence showed lower thresholds for visceral discomfort and pain in the majority of patients with IBS [48,49,50]. It has been shown that WA is an effective model of inducing VH in animals [33,51]. Similar to the previous experiment, there was no statistical difference between the groups in terms of weight and feed consumption in the present study, eliminating other factors affecting VH. However, the mechanism underlying VH in IBS is complex. It is now believed that intestinal barrier destruction, abnormal activation of silent gastrointestinal nociceptors, peripheral intestinal nerves, and central nervous system sensitization are also involved [52,53]. Here, we found that VH significantly correlated with colonic microcirculation.

To the best of our knowledge, the present study is the first to reveal the changes in colonicmicrocirculation in an IBS mouse model and directly after FODMAP diet intervention. Two potential central mechanisms may support these results. First, regional intestinal blood-flow-regulating factors affect intestinal blood flow. Second, changes in blood flow result from altered autonomic nervous system function and neurotransmitters. In this experiment, we confirmed that there was no statistical difference in the blood flow of the mesenteric artery supplying the proximal colon among the groups, which suggests that regional intestinal segment lesions caused changes in the blood flow of the colonic microcirculation. Nevertheless, the causal relationship between colonic microcirculation reduction and IBS pathogenesis in the WA-HF group remains to be determined. Interestingly, this phenomenon can be ameliorated by a low-FODMAP diet by dilating microcirculation and improving blood infusion.

VEGF is a powerful factor in vasculogenesis and angiogenesis processes in both development and pathological conditions, mainly targeting endothelial cells; it is also known as VEGF-A [54]. Moreover, VEGF is considered an inflammatory factor that can affect vascular permeability and regulate the recruitment of inflammatory cells in colitis diseases [55]. Low-grade mucosal inflammation is considered one of the causes of IBS [56]. Here, we verified that the expression of VEGF protein was significantly higher in the HF group and significantly reduced in the LF group, indicating that the number of microvessels might increase in the HF group. A small-sample study showed that the serum VEGF level in IBS patients was higher than in ordinary people [57]. Another double-blinded randomized trial discovered that the severity of IBS symptoms in patients decreased, and circulating levels of proinflammatory cytokines, including IL-6, IL-17, and interferon-γ were significantly reduced, as well as VEGF, after turanicum wheat dietary intervention [58]. Christina et al. focused on the treatment of stress-related and psychological IBS. They found that after the one-year experience with choirs, VEGF levels were higher in both choir and nonchoir groups [59]. Experiments in other tissues confirmed that in the brain and retina, VEGF signaling is closely related to leukocyte adhesion and capillary blockage [60,61]. Nevertheless, there remains a knowledge gap associated with the mechanism of the VEGF pathway signaling and tissue microcirculation, and more research needs to be conducted.

IBS is believed to be linked to disruptions in autonomic nervous system regulation, which can damage vascular homeostasis and cause IBS symptoms and exaggerated responses to stress [62,63]. Tanaka et al. measured finger blood perfusion using laser doppler blood flow, an indicator of sympathetic nerve function. It was found that the blood flow response of IBS patients decreased, indicating excessive sympathetic nerve activity [64]. A separate study showed that rectal blood flow was decreased in IBS due to excitation of the extrinsic colonic vagus nerve and a deficit in cholinergic activity [65]. Based on this evidence, it is reasonable to speculate that sympathovagal imbalance or dysfunction may result in altered colonic microcirculatory flow in IBS patients.

Currently, diet management is considered one of the most critical decisions in IBS management strategies, especially FODMAP food restriction [1]. The research has shown that FODMAP aggravates VH by increasing the water volume in the small intestine and gas production in the colon [14]. Zhou et al. revealed that a FODMAP diet modulates barrier dysfunction, thus activating visceral pain [18]. In the meantime, too much gas and/or water fermented by intestinal bacteria can lead to an intraluminal pressure increase. Boley et al. showed that colonic blood flow decreased stepwise with increasing cavity pressure [66], although the mesenteric blood pressure remained unchanged. Different diameters of the local intestinal cavities lead to a stretch of the intestinal cavity at the expansion site [67], which may potentially induce abdominal pain. In the present study, the threshold for VH in the WA-HF group was lower than that in the WA-RF group. However, we did not observe that colonic microcirculation in the WA-HF group was less than that in the RF group.

This study had several limitations. First, the results of this study are limited to a mouse model of WA stress, although it is a classic model simulating the preclinical experiment of IBS. The mechanism underlying low FODMAP requires further investigation in clinical practice. Second, the side effects of FODMAP in the body require further research and confirmation. Third, the study’s investigation of intestinal microcirculation focused on the colon. Further research is required to determine the effects of different doses of FODMAP on microcirculation in other regions of the intestine.

## 5. Conclusions

Our study showed that a low-FODMAP diet could alleviate VH by improving colonic microcirculation. Colonic microcirculation blood flow was positively correlated with the threshold of VH. This may provide a theoretical basis for limiting the FODMAP diet for IBS management.

## Figures and Tables

**Table 1:** Composition of the custom diets (g/kg).

Raw Material	Regular FODMAP (2.1%)		High FODMAP (10%)		Medium FODMAP (5%)		Low FODMAP (0%)	
	Weight (g)	Energy (Kcal)	Weight (g)	Energy (Kcal)	Weight (g)	Energy (Kcal)	Weight (g)	Energy (Kcal)
Casein	200	800	200	800	200	800	200	800
Cystine	3	12	3	12	3	12	3	12
Soybean oil	70	630	70	630	70	630	70	630
Lard	0	0	0	0	0	0	0	0
Sucrose	100	400	100	400	100	400	100	400
Maltodextrin	132	528	132	528	132	528	132	528
Corn starch	376	1504	297	1188	347	1388	397	1588
Cellulose	50	0	50	0	50	0	50	0
Multimineral ain	35	0	35	0	35	0	35	0
Multidimensional	10	40	10	40	10	40	10	40
Choline	2.5	0	2.5	0	2.5	0	2.5	0
Fructose	7.35	29.4	35	140	17.5	70	0	0
Fructo-Oligosaccharide	6.3	25.2	30	120	15	60	0	0
Galacto-oligosaccharide	7.35	29.4	35	140	17.5	70	0	0
Sorbitol	0	0	0	0	0	0	0	0
Total	999.5	3998	999.5	3998	999.5	3998	999.5	3998
	Weight ratio	Energy ratio	Weight ratio	Energy ratio	Weight ratio	Energy ratio	Weight ratio	Energy ratio
Protein	0.203101551	0.203101551	0.203101551	0.203101551	0.203101551	0.203101551	0.203101551	0.203101551
Fat	0.070035018	0.157578789	0.070035018	0.157578789	0.070035018	0.157578789	0.070035018	0.157578789
Carbohydrate	0.63931966	0.63931966	0.63931966	0.63931966	0.63931966	0.63931966	0.63931966	0.63931966

## Data Availability

Not applicable.
